# Health Tract No. 315

**Published:** 1868-03

**Authors:** 


### HEALTH TRACT No. 315.

### PRESENTIMENTS

Are impressions made upon the mind which seem to foreshadow some future event, and the general feeling appears to be that a Divine Influence is involved. According to general received opinion, Aaron Burr was the worst and most wicked and profligate man of his age. His perents and grandparents were among the very best in intellect, learning and piety in their century and yet his grandmother, the wife of the immortal Edwards Jonathan wrote a few days after his birth, "A strange presentiment of evil has hung over my mind of late, and I can hardly rid myself of the impression that that child is born to see trouble, for lately I had a sad night of broken sleep, in which the future career of that boy seemed to pass before me. He first appeared as a little child, just beginning to ascend a high hill. Not long after he set out, the two guides who started with him disappeared, one after the other. He went on alone, and as the road was open and plain, and as friends met him at every turn he got along very well. At times he took on the air and bearing of a soldier, and then of a statesman, assuming to lead others. As he neared the top of the hill, the way grew more steep and difficult and his companions became alienated from him, refusing to help him or to be led by him. Baffled in his designs, and angered at his ill-success, he began to lay about him with violence, leading some astray, and pulling down others at every attempt to rise. Soon he himself began to slip and slide down the rough and perilous sides of the hill; now regaining his foothold for a little, then losing it again, until at length he stumbled and fell headlong down, down, into a black and yawning gulf at the base!

"At this, I woke in distress, and was glad enough to find that it was only a dream. I think the disturbed state of our country, along with my own indifferent health, must have occasioned it. A letter from his mother to-day, assures me that her little Aaron is a lively, prattlesome fellow, filling his parent's hearts with joy."

President Lincoln had a presentiment immediately preceeding his assassination in the repetition of a fearful dream for the third or fourth time, and which in each case was followed by a great national calamity.

At midnight before the battle of Wagram, Lascelles asked Napoleon to sign the degree for his title, to be transmissible to his son, because he felt that he would fall in the next day's battle; and he did. "Here I am," said Carroin to Napoleon at Echmul, as the Emperor had sent for him from Marseilles, "but this is my last day;" a quarter of an hour afterwards a cannon ball took off his head. Pilates' wife was impressed by a dream several times repeated of some impending calamity, and warned her husband not to have anything to do "with that just man;" while volumes may be filled with "presentiments" more or less justified by succeeding events.

But how many millions of presentiments have not been verified? How many times has the reader himself suffered from a sense of impending calamity, without ever having had a verification of the same?

How many terrible dreams have been followed by days which have had not a ripple on life's surface. In nine cases out of ten, what's the use of a presentiment? it does not lead to any change of conduct, does not result in any practical good.

President Lincoln made no saving use of his terrible dream so oft repeated. Aaron Burr's grandmother does not seem to have taken any measures to forestall the purport of her dream. Revelations ceased with the Savior's age; all was said then, that could have been said to man's advantage, and he himself said as to the practical use of further revealings; "If they hear not Moses and the Prophets, neither will they be persuaded though one rose from the dead." We cannot suppose that the Ruler of the Universe would step out of his path in order that a murderous infidel French officer should have a son who should be called general Frappeau, instead of Johnson Frappeau.

Many feel that the first "Bull Run" was in reality among the greatest blessings of the war. In that case the President had a presentiment that was not a presentiment. Besides, why should we not have presentiments of good rather than of evil, as the Infinite One is the embodiment of benevolence. No doubt if pigs could talk, they would entertain us by the hour with the hugest presentiments of modern times, enough to fill Bonner's Ledger with Hobgoblins for a year. The truth is, people who have good health don't have presentiments, which are really the offspring of idleness and gluttony, of an overloaded stomach, of a brain fed with bad blood, for that innumerable multitude who over-eat every day, have neither good blood, good sense or a good conscience.

